# Evaluation of the relationship between clinical and laboratory risk factors in atherosclerosis patients with coronary slow flow: a case–control analysis

**DOI:** 10.1186/s43044-023-00388-9

**Published:** 2023-07-13

**Authors:** Reza Faramarzzadeh, Farin Fekrat, Arian Haghtalab

**Affiliations:** 1 Department of Cardiology, Ayatollah Taleghani Hospital, Kashani St., Urmia, 5715974677 West Azerbaijan Iran; 2 Faculty of Medicine, Urmia University of Medical Sciences, Urmia, West Azerbaijan Iran

**Keywords:** Risk factor, Slow coronary flow, CSF, CSFP, Inflammation

## Abstract

**Background:**

Coronary slow flow (CSF) is an angiographic entity distinguished by the delayed filling of the epicardial coronary arteries in the lack of significant obstructive artery disease. The pathological causes are still unknown. This study aimed to elucidate the relationship between clinical and laboratory-related risk factors in atherosclerosis patients diagnosed with CSF.

**Results:**

The research encompassed a study group of 142 individuals, with a mean age of 52.47 ± 10.62, and a male representation of 47.7%. A thorough statistical analysis was conducted, indicating that there were no noteworthy variations in age, gender, smoking history, hematocrit, blood sugar, and HDL levels between the groups of cases and controls (*P* > 0.05). Subsequent analysis of the data indicated that there were significant differences in history of hypertension, LDL, and BMI measurements between the groups of subjects who were designated as cases and those who were designated as controls. Our study revealed that male gender, a history of hypertension, and BMI were identified as independent predictors of CSF (*P* < 0.05).

**Conclusions:**

After modeling regression, we were able to conclude that male gender, BMI, and history of hypertension are reliable predictors of slow coronary flow. These findings add to our growing understanding of the complex interplay between clinical and laboratory risk factors in the development and progression of CSF.

## Background

The slow coronary flow (CSF) phenomenon, also called cardiac syndrome Y, is described as the lagged opacification of the coronary vessels at the distal portion. The diagnosis of CSF is mainly based on the delay in the release of the contrast agent during angiography. The phenomenon occurs while the patient does not have coronary artery disease (CAD) and represents a disorder in coronary microvascular resistance, resulting in abnormal, slow coronary flow. It is reported in 1–7 percent of patients undergoing diagnostic coronary angiography [1, 2]. Tambe et al. (1972) first described this syndrome, and since then, it has become known as an independent syndrome called CSF, primary coronary slow flow, coronary slow flow, or cardiac syndrome Y. The prefix primary is used to distinguish it from cases where the symptom occurs for secondary causes (e.g., cardiac reperfusion treatments like angioplasty or stent placement for acute myocardial infarction (MI)) [3–5]. Angiograms of patients with CSF are usually interpreted as slow flow coronary arteries [6, 7]. The clinical course of the disease is complex and lacks a unique clinical feature. According to some studies, this syndrome may have direct clinical complications beyond being only a clinical finding during angiography. More than 80% of patients encounter recurrent chest pain, characterized by angina pectoris, and 20% of patients require hospitalization [8]. This phenomenon is associated with some risk factors for coronary heart disease, such as high body mass index and smoking [9]. Increased pulse pressure have been reported in patients. Moreover, mitochondrial changes, low glycogen levels in myositis, and hypertrophy have been reported among CSF cases [10, 11]. Although the syndrome has been known for almost 50 years, its pathogenesis and risk factors that make a person susceptible to the disease are still not properly known. Studies on the risk factors influencing the disease have yielded diverse results. Decreased epithelial progenitor cells [12], high body mass index (BMI) and male gender [13], male gender and obesity [14], blood pressure, dyslipidemia, and history of smoking [15] have been reported as risk factors of the condition. It is believed that some metabolic, genetic, and other factors, along with microvascular disorders, endothelial tissue dysfunction, atherosclerosis, and inflammatory processes, may have a role in the pathogenesis of this syndrome [9]. The syndrome generally affects patients' daily lives by causing direct cardiovascular complications and severely reducing their quality of life. However, there are a few clinical studies of CSF. Physicians less consider the syndrome, treatment, and its cardiac complications. The probability of the syndrome incidence is even underestimated. Hopefully, this study will help better understand CSF risk factors, offer innovative prevention strategies for susceptible subjects, and take a step toward relieving some disease burden.

## Methods

### Patient selection

The present study, which follows a case–control design, was carried out at Ayatollah Taleghani Hospital located in Urmia, Iran. The study participants were individuals who had undergone coronary angiography, and the necessary data were retrieved from an electronic database with the appropriate consent from local authorities. Individuals exhibiting the subsequent traits were selected for further scrutiny: The study included individuals who met the following criteria: (1) age over 18 years; (2) presence of acute coronary syndrome, stable angina, or atypical chest pain upon admission; and (3) confirmation of normal coronary arteries through angiography. Upon examination of the coronary angiography outcomes, a total of 152 patients were selected for the study, with 76 patients assigned to both the control and case groups.

### Frame counting

The TIMI frame count is determined by the duration of substance transit to the luminal surface of the coronary arteries. The determination of distances within the coronary arteries is established by identifying specific landmarks. This information is then utilized to compute and define the duration necessary for the contrast material to reach a particular point. This process enables consistent imaging of the contrast medium's progression through the vessel. The aforementioned visual representation was recorded at a frequency of 30 frames per second. The outcome is presented as the count of frames necessary for the contrast agent to traverse. The present investigation employed the TIMI frame count technique, as outlined in Gibson's study [16], to ascertain the cohort of interest. In order to quantify the frames, the origin was established as the initial position that fully saturated the color of the arterial outset. The terminal point of chromaticity on a given branch was designated as the final frame. The study employed a frame counter to ascertain the quantity of cine frames necessary for the attainment of standard distal coronary landmarks in the left anterior descending, left circumflex, and right coronary arteries. According to the research conducted by Gibson [16] a frame count exceeding 27 was identified as indicative of coronary slow flow.

### Data extraction

Data on demographic variables related to age and gender were gathered. Data pertaining to the conventional risk factors for coronary artery disease, namely hypertension, diabetes, dyslipidemia, and smoking, was gathered. The study documented the BMI, measurements of blood pressure, blood sugar (BS) levels, lipid profile, and hematocrit values during the time of coronary angiography for all participants.

### Data analysis

The chi-square test was employed to analyze categorical variables, where appropriate. The independent *t*-test or Mann–Whitney *U* test was employed to analyze continuous variables. The utilization of logistic regression analysis was employed to evaluate the factors that predict slow flow. The study employed a multivariate logistic regression analysis model to calculate the respective odds ratios (OR) with 95% confidence intervals (CI). The statistical analysis of the data was conducted using SPSS 26, which is a widely used software package for social science research.

## Results

According to the results obtained from the univariate analysis (as presented in Table 1), the average age of the entire population was 52.47 ± 10.62. The study computed the mean age of the case and control groups to be 52.42 ± 10.78 and 52.51 ± 10.53, respectively. The utilization of the independent parametric *t*-test was necessitated by the normality of the age distribution in the study population, as confirmed by the Kolmogorov–Smirnov test, in order to compare the groups. The calculated independent parametric t-test value of 0.95 indicates that there was no significant difference observed between the mean ages of the two groups.

**Table 1:** Comparison of two case–control groups in terms of demographic findings, comorbidities, laboratory measurements, and angiography indications

	Control (*n* = 76)	Case (*n* = 76)	*P* value
Demographics			
Age	52.51 ± 10.53	52.42 ± 10.78	0.95
Male	40.8%	52.6%	0.19
Female	59.2%	47.4%	
Comorbidities			
BMI	26.04 ± 2.72	27.90 ± 5.92	0.005*
History of HTN	26.3%	44.7%	0.02*
History of CAD	14.5%	22.4%	0.29
Smoking	9.2%	13.2%	0.45
Measurements			
BS	102.43 ± 35.52	104.7 ± 39.46	0.78
LDL	74.36 ± 21	81.24 ± 22.51	0.05*
HDL	39.52 ± 7.94	39.81 ± 9.13	0.83
Hct	39.45 ± 3.89	39.60 ± 3.70	0.74
Angiography indication			
ACS	43.4%	43.4%	0.3
UA	44.7%	35.5%	
Positive exercise stress test	7.9%	13.2%	
Changes in ECG	0%	1.3%	
CHF	3.9%	3.9%	
IHD	0	2.6%	

Results denoted with an asterisk (*) exhibit those with statistical significance

Among the cohort of 152 patients, 71 individuals (46.7%) were identified as male, while 81 individuals (53.3%) were identified as female. Within the case group, the gender distribution was as follows: 40 individuals (52.6%) were male, while 36 individuals (47.4%) were female. The control group comprised of 31 cases (40.8%) that were male and 45 (59.2%) that were female. The Chi-square test was employed to conduct a comparison of the groups based on gender. The results indicated that there was no significant difference between the groups (*P* = 0.19).

The mean BMI of the entire sample population was 26.97 ± 4.07 kg/m^2^. Our findings indicate that the average BMI in the case group was 27.90 ± 5.92 kg/m^2^, while in the control group, it was 26.04 ± 2.72 kg/m^2^. We employed the Kolmogorov–Smirnov test to assess the nature of the BMI distribution. The test yielded a result exceeding 0.05, indicating that the BMI distribution in the population under investigation was normal. Consequently, we utilized the independent t-test to compare the mean BMI values between the case and control groups. The results of the analysis revealed a statistically significant disparity between the case and control groups, suggesting that the mean BMI of the case group was greater than that of the control group (*P* = 0.005).

Out of the total cases examined, 54 cases (35.5%) had a medical history of hypertension, 17 cases (11.2%) had a history of smoking, and 28 patients (18.4%) had a medical history of coronary artery disease. The case group exhibited a prevalence of hypertension history in 34 patients (44.7%), while the control group had a lower prevalence of 20 patients (26.3%). The statistical method employed to compare the groups was the Chi-square test. The results indicate a significantly higher incidence of hypertension in the case group (*P* = 0.02), while no significant differences were observed between the groups in terms of smoking history (*P* = 0.45) and history of coronary artery disease (*P* = 0.29).

The study population's mean values for BS, LDL, HDL, and hematocrit were calculated as follows: 103.25 ± 37.43 mg/dL, 77.78 ± 21.96 mg/dL, 39.66 ± 8.52 mg/dL, and 39.56 ± 3.78, respectively. The mean values of BS, LDL, HDL, and hematocrit in the case and control groups are presented in Table 1. The utilization of an independent t-test was necessitated by the normal distribution of variables in the study population, in order to compare the mean values between the groups. The results indicate that the mean LDL levels were significantly higher in the case group compared to the control group (*P* = 0.05). However, there were no statistically significant differences observed between the case and control groups in terms of BS (*P* = 0.78), HDL (*P* = 0.83), and hematocrit levels (*P* = 0.74).

Angiography was primarily performed for 66 patients (43.4%) due to acute coronary syndrome (ACS). The Chi-square test was utilized to conduct a comparison of angiography between the case and control groups. The results indicated that there was no significant difference between the two groups (*P* = 0.3).

According to Table 2, left anterior descending (LAD) was the most common vessel involved (97.4% of the patients), and in most of the patients only one vessel was involved (47.4%).

**Table 2:** Evaluation of CSF patients in terms of the type of involved vessel and the number of involved vessels

	Frequency	Percentage
LAD		
Involved	74	97.4
Not involved	2	2.6
LCX		
Involved	38	50
Not involved	38	50
RCA		
Involved	21	27.6
Not involved	55	72.4
Number of involved vessels		
1	36	47.4
2	22	28.9
3	18	23.7

According to the findings of the multivariate study (Table 3), male gender (OR = 0.23, 95% CI: 0.09–0.61, and *P*-value = 0.003), history of hypertension (OR = 4.40, 95% CI: 1.68–11.52, and *P*-value = 0.002), and high BMI (OR = 0.82, 95% CI: 0.73–0.92, and *P*-value = 0.001) were found to be the factors that predicted CSF incidence.

**Table 3:** Odds ratio and its confidence interval in having slow flow of coronary arteries for the studied variables

Variable	Variable description	Odds ratio	CI 95%	*P*-value
Age	–	1.003	0.96–1.04	0.87
Gender	Male	0.23	0.09–0.61	0.003*
	Female	–	–	–
History of HTN	No	–	–	–
	Yes	4.40	1.68–11.52	0.002*
History of CAD	No	–	–	–
	Yes	1.05	0.45–3.52	0.66
Smoking	No	–	–	–
	Yes	1.7	0.49–5.87	0.39
BMI	–	0.82	0.73–0.92	0.001*
BS	–	1	0.99–1.01	0.93
LDL	–	0.98	0.96–1.00	0.14
HDL	–	0.99	0.93–1.00	0.42
Hct	–	0.94	0.94–1.17	0.38

Results denoted with an asterisk (*) exhibit those with statistical significance

## Discussion

This study examined the correlation between risk factors and clinical and laboratory factors in patients with atherosclerosis and CSF. The study included 46.7% males and 53.3% females, with an average age of 52.47 ± 10.62 years. No significant differences were found between the groups based on gender (*P* = 0.19) or age (*P* = 0.95). However, logistic regression analysis indicated that male gender predicted CSF in our study (*P* = 0.003).

This study's results align with Sanghvi et al.'s [15] study, which also found a higher occurrence of CSF in males. However, a significant gender difference between case–control groups was found (*P* = 0.012) in the study by Sanghvi [15], which contrasts with the current study's results (*P* = 0.19). Li et al. [9] reported similar findings to ours, with no significant differences in age or gender between the control and case groups. Hawkins et al. [14] found no significant age or gender differences between groups but confirmed that male gender predicts CSF incidence through regression analysis.

The statistical analysis conducted in the current study revealed that hypertension was more commonly observed in individuals with CSF as compared to those without the condition (*P* = 0.02). Also, logistic regression analysis indicated that hypertension predicted CSF in our study (*P* = 0.002). But, there were no statistically significant variations detected between the groups with regards to their smoking history and the presence of coronary artery disease (*P* = 0.45 and 0.29, respectively). The study conducted by Li et al. [9] did not reveal any noteworthy correlation between smoking and CSF (*P* = 0.033). Additionally, there was no significant variance observed in blood pressure levels between the groups (*P* = 0.08 for systolic blood pressure and *P* = 0.79 for diastolic blood pressure). The study conducted by Sanghvi et al. [15] revealed a statistically significant variation in the prevalence of hypertension between the case and control groups (*P* < 0.001). Additionally, the authors identified hypertension as a predictor of CSF (*P* < 0.001). The smoking prevalence among the case group was found to be significantly higher than that of the control group (*P* < 0.001), which is in contrast to the findings of our study. According to Sanghvi et al.'s findings, smoking was identified as a predictor of CSF with a significance level of *P* = 0.001. The study conducted by Hawkins et al. [14] revealed that there was no statistically significant variation in nicotine consumption between the case and control groups (*P* = 0.68). Furthermore, hypertension was not acknowledged as a predictor of CSF.

The current investigation examined several laboratory factors, including blood sugar, hematocrit, LDL, and HDL. The findings revealed a noteworthy dissimilarity in LDL levels between the two groups (*P* = 0.05), with the case group exhibiting elevated LDL levels. Nevertheless, the study results indicate that there were no statistically significant variations in the levels of blood glucose, hematocrit, and HDL (with corresponding *P*-values of 0.78, 0.74, and 0.83, respectively). Li et al. [9] observed that there were no statistically significant variations in the levels of LDL and HDL between the case and control groups (*P* = 0.489 and 0.292, respectively). This outcome is in line with the current study's results for HDL, but not for LDL. In their study, Yilmaz et al. [17] found a statistically significant correlation between LDL levels and CSF in patients, indicating that patients with CSF had higher levels of LDL. A notable variation in fasting blood glucose levels was observed between the groups, which contrasts with the outcomes of the current investigation. The study conducted by Mukhopadhyay et al. [18] did not yield a statistically significant distinction in hematocrit percentage among the various groups.

In the current study, the independent predictive value of body mass index (BMI) for CSF was demonstrated (*p* = 0.001), which is consistent with the findings that were reported by Hawkins et al. (*p* 0.01) [14] and Mukhopadhyay et al. (*p* = 0.003) [18]. In addition, Li and colleagues [9] found that there was a significant correlation between BMI and CSF (*p* = 0.013).

## Conclusions

The results showed that hypertension and higher levels of serum LDL concentration were significantly higher in patients with CSF. Regression analysis also suggested that male gender and hypertension can be independent CSF predictors.

### Study limitations

Due to the differences between the results in the literature and the findings of the present study, broader research with large sample size and different and comprehensive models (e.g., a cohort study) is recommended to understand CSF causes and factors better.

## Data Availability

The data that support the findings of this study are available from Urmia University of Medical Sciences and Ayatollah Taleghani Hospital of Urmia but restrictions apply to the availability of these data, which were used under license for the current study, and so are not publicly available. Data are however available from the authors upon reasonable request and with permission of Urmia University of Medical Sciences and Ayatollah Taleghani Hospital of Urmia.

## Abbreviations

ACS: Acute coronary syndrome
BMI: Body mass index
BS: Blood sugar
CAD: Coronary artery disease
CHF: Congestive heart failure
CSF: Coronary slow flow
ECG: Electrocardiogram
FBS: Fasting blood sugar
HDL: High-density lipoprotein
HTN: Hypertension
IHD: Ischemic heart disease
LAD: Left anterior descending
LCX: Left circumflex
LDL: Low-density lipoprotein
MI: Myocardial infarction
RCA: Right coronary artery
SPSS: Statistical package for the social science
TIMI: Thrombolysis in myocardial infarction
TG: Triglyceride
UA: Unstable Angina
